# Organ donation after euthanasia starting with anesthesia at home is legal in The Netherlands, Belgium, Canada and Spain

**DOI:** 10.1186/s12910-023-00906-z

**Published:** 2023-05-29

**Authors:** Johannes Mulder, Hans Sonneveld

**Affiliations:** Anaesthesiology-Intensive Care Department, Isala Hospitals, Zwolle, The Netherlands

**Keywords:** Euthanasia, Organ donation, Organ donation after euthanasia, Organ donation after euthanasia from home, Awareness, Anesthesia, Autonomy, Legal

## Abstract

We would like to respond to the article “Organ donation after euthanasia starting at home in a patient with multiple system atrophy Tajaâte et al., [2021] 22:120” on organ donation after euthanasia from home [ODEH]. Although we welcome the performance of ODEH, we would like to make some critical comments regarding the article, both in relation to factual inaccuracies and in terms of the vision expressed on this subject. In this letter we stress the protection of autonomy of vulnerable euthanasia patients, we contradict the assumption of illegality, we question if the anesthesia method utilized is optimal and correct a mistake in regard to an article to which is referred of ourselves.

## Letter to the editor/correspondence

**Dear Editor**

We welcome the article of Tajaâte et al. on organ donation after euthanasia from home [ODEH] which will continue to raise awareness of the opportunity for a specific group of euthanasia patients to pass life as a gift [by donating organs] to others while they lay down their lives [1]. This is the third publication on ODEH, following publications by Healey et al. and Mulder et al. [2, 3]. The classical “Organ donation after euthanasia [ODE]” procedure offered over 200 patients who wished for ODE, the option in a careful, legal and considered manner [4–6]. ODEH accommodates the significant objection of ODE for the patient to go to hospital in a conscious state by allowing the patient to enter complete loss of consciousness in the comfort of their own home [7]. During the recent first ODE[H] conference held in 2021 this advantage was recognized and supported by all participants from performing countries [6].

We have some comments on the article by Tajaâte et al. The abstract states that “patients are nevertheless willing to help others”. It implies that suffering euthanasia patients are actively asked to cooperate with organ donation. Supported by the Dutch and Canadian guidelines, we take the view that the ethical principle must be that the addition of organ donation to euthanasia must be patient-initiated for protection of autonomy reasons with the vulnerable euthanasia patients [4, 8, 9]. Consequently, it would be better to say that patients are asking, rather than willing, to help others.

The assertions concerning the legality of ODEH are concerning and likely to give rise to unnecessary disquiet. Under Dutch law, the euthanasia provider must be convinced that the due diligence requirements (also known as appropriate care or due care requirements) are satisfied prior to the start of the euthanasia procedure. The procedure itself consists of the elements premedication, medical coma induction and muscle relaxation, as stated in the Dutch euthanasia guideline. Claiming that due diligence requirements are no longer satisfied once the euthanasia procedure has started, irrespective of the time frame, is irrational. This is also confirmed in the three ODEH cases by the Dutch Regional Euthanasia Review Committees [10, 11].

We also question the described sedation method “general anesthesia was induced with 15 mg midazolam, 10 mg piritramide (a synthetic morphine analog, equal to 7,5 mg morphine) and 100 mg rocuronium”, following which intubation, ventilation, transportation, abdominal ultrasound examination, intra-arterial line insertion took place before the euthanasia procedure was concluded. Despite the reference to “general anesthesia”, this method does not appear to include an anesthetic [such as propofol/thiopental etc.], even though the risks of awareness [while the patient is fully paralyzed] where only a sedative is used are well-known [12].

It is also stated that the difference between their procedure and an earlier ODEH case was that death took place close to the operating theatre “for the best organ quality”, accompanied by a reference to Mulder et al. [3]. This is also incorrect. The referred to, first ODEH case from 2017 was also concluded close to the operating theatre, with a warm ischemia time of only 7 min for optimal organ quality [3, 11].

Finally, although it is correct that the official ODE guideline [11] has not yet adopted a formal position on ODEH, the national Health Council has stated its position at the request of the Minister of Health: “The committee regards the procedure in principle as a viable route, provided that it does not impede a careful establishment of death” [13]. This is also true for Canada were the first ODEH cases are performed [2]. Furthermore, the option is mentioned in the 2022 revised version 3 of the ODE guideline [14].

## Data Availability

Not applicable.

## Abbreviations

ODE: Organ donation after euthanasia
ODEH: Organ donation after euthanasia from home
